# Intensive care unit professionals’ responses to a new moral conflict assessment tool: A qualitative study

**DOI:** 10.1177/09697330231151352

**Published:** 2023-05-25

**Authors:** Soodabeh Joolaee, Deborah Cook, Jean Kozak, Peter Dodek

**Affiliations:** Center for Health Evaluation and Outcome Sciences, St Paul’s Hospital and University of British Columbia, Vancouver, BC, Canada;; Fraser Health Authority, Surrey, BC, Canada; Nursing Care Research Center, Iran University of Medical Sciences, Tehran, Iran; Department of Medicine, McMaster University, Hamilton, ON, Canada; Department of Family Medicine, 102794 Providence Health Care, Vancouver, BC, Canada; Division of Critical Care Medicine and Center for Health Evaluation and Outcome Sciences, 12358 St Paul’s Hospital and University of British Columbia, Vancouver, BC, Canada

**Keywords:** Moral distress, intensive care unit, moral conflict assessment, qualitative

## Abstract

**Background:**

Moral distress is a serious problem for health care personnel. Surveys, individual interviews, and focus groups may not capture all of the effects of, and responses to, moral distress. Therefore, we used a new participatory action research approach—moral conflict assessment (MCA)—to characterize moral distress and to facilitate the development of interventions for this problem.

**Aim:**

To characterize moral distress by analyzing responses of intensive care unit (ICU) personnel who participated in the MCA process.

**Research Design:**

In this qualitative study, we invited all ICU personnel at 3 urban hospitals to participate in individual or group sessions using the 8-step MCA tool. These sessions were facilitated by either a clinical ethicist or a counseling psychologist who was trained in this process. During each session, one of the researchers took notes and prepared a report for each MCA which were analyzed using qualitative content analysis.

**Participants and Research Context:**

A total of 24 participants took part in 15 sessions, individually or in groups; 14 were nurses and nurse leaders, 2 were physicians, and 8 were other health professionals.

**Ethical Considerations:**

This study was approved by the Providence Health Care/University of British Columbia Behavioural Research Ethics Board. Each participant provided written informed consent.

**Results:**

The main causes of moral distress related to goals of care, communication, teamwork, respect for patient’s preferences, and the managerial system. Suggested solutions included communication strategies and educational activities for health care providers, patients, family members, and others about teamwork, advance directives, and end-of-life care. Participants acknowledged that using the MCA process helped them to reflect on their own thoughts and use their moral agency to turn a distressing situation into a learning and improvement opportunity.

**Conclusions:**

Using the MCA tool helped participants to characterize their moral distress in a systematic way, and to arrive at new potential solutions.

## Introduction

Moral distress is a serious problem for health care personnel. Measurement of this problem is essential as a foundation for action. However, surveys, individual interviews, and focus groups may not capture all of the effects of, and responses to, moral distress. Therefore, we used a new participatory action research approach—moral conflict assessment (MCA)—to characterize moral distress and to prompt specific action for this problem.

## Background

Moral distress, which has been associated with negative outcomes in both health care providers and patients, was first defined by Jameton in 1984 as “the consequence of being constrained from moving from moral choice to moral action”.^1^ Moral choice refers to the decision to act according to what an individual believes to be correct, and moral action refers to making that decision a reality. Initially focused only on nurses in intensive care settings, subsequent research has both refined the definition of moral distress as well as expanded it to include other health and allied health professionals who provide care in various clinical environments and also experience the impact of moral distress on themselves, their colleagues, and their patients.^2–7^ Specifically, in response to moral distress, health care professionals experience a variety of negative emotions, are prone to consider quitting their jobs, and they perceive that patient care is negatively affected.^8^

Moral distress is common in intensive care unit (ICU) personnel,^2,4,9,10^ and the consequences of this distress may be deleterious to the individual experiencing it, to others, and to the health care system. Effects at the individual level include anger towards one’s self, self-doubt, diminished self-esteem, depression, and burnout. Effects directed towards others include anger, incivility, bitterness, cynicism, dismay, and frustration. Effects on the health care system include avoidance behaviors, leaving the workplace, and leaving the profession.^10^

The most commonly used tool to assess moral distress is a survey that was developed and revised by Corley^3^ and Hamric.^9^ However, the standard approach of using surveys, interviews, and focus groups may not fully characterize moral distress in individuals and teams.^10^ In addition, these approaches do not guide affected individuals to develop new adaptive responses to situations that cause moral distress. Therefore, we used a participatory action research approach to develop a new MCA tool to further characterize moral distress and to facilitate the development of interventions for this problem.^11,12^

## Aim

The aim of this study was to characterize moral distress by analyzing responses of ICU personnel who participated in the MCA process.

## Research design

We used a qualitative content analysis to analyze responses of ICU personnel who participated in this MCA process.

The 8-step MCA tool was developed by Chevalier and colleagues as an explicit tool to assist researchers and participants to reflect on experiential knowledge and to synthesize this knowledge into action for improvement.^11,12^ It is also a source of data about causes, consequences, and solutions related to moral distress. Briefly, in step 1, participants decide whether they will proceed with this assessment as individuals or as a group, and if they will include a facilitator. In Steps 2, 3, and 4, they describe what they have been constrained to do that is causing moral conflict, the effects on them, and their responses, respectively. Step 5 identifies the core values, self-interests, and elements of identity that are either coherent or in conflict with the constraining behavior. Step 6 identifies existing factors that help or hinder participants’ ability to address the situation. A review of all of the preceding steps informs a coping strategy and feasible action plan to remedy the situation and cope better (Step 7). Finally, participants state how they feel about their moral conflict after completing this assessment, and if the effect it has on them has changed in any way (Step 8).

Each assessment was facilitated by one of two clinical ethicists or a counseling psychologist who were trained in this process and who were employees at the institutions that participated in this study. None of these facilitators had any previous relationships with the researchers. However, given the participatory nature of the research, they were considered part of the research team for this work. At the beginning of the MCA sessions, the concept of moral distress was described to participants and the process and steps of the tool were explained. The interview team which was composed of the facilitator and a clinician/researcher who had experience in critical care then used directed questions to elicit responses from participants for each step of the MCA tool. During each session, the researcher took detailed notes and prepared a report for each MCA. No audiotaping or videotaping was done because of the sensitive nature of the conversation about each individual’s workplace and because the format of the MCA is to complete items and answer questions with short phrases or single words (the MCA tool is designed to be completed by a participant or group of participants without a facilitator). Each session lasted about 1 hour. To assure the accuracy of data, before each report was finalized, the researcher’s notes and direct quotations were checked and confirmed by the respective participants.

## Participants and research context

We invited all ICU personnel at 3 hospitals in the greater Vancouver area (1 tertiary and 2 large community hospitals) to participate. This invitation was sent by email from the leader of each ICU. Individual and group interviews were completed between July 2017 and April 2018. Between July 2017 and April 2018, a total of 24 participants from the 3 hospitals took part in 15 sessions, individually or in groups; 14 were nurses and nurse leaders, 2 were physicians, and 8 were other health professionals (from pharmacy, nutrition, spiritual care, and social work).

## Data analysis

All reports of MCAs were analyzed using a qualitative content analysis, which was done by an experienced qualitative health researcher (SJ). The key feature of all content analysis is that the many words of the text are classified into much smaller content categories called codes^13–15^ and there is minimal interpretation. The preliminary coding scheme was created based on the 8 steps of the MCA tool. To get a better sense of the data, we read the reports through and manually coded participants’ responses to each item in the MCA tool by highlighting the exact words from the text that appear to capture critical thoughts or concepts. Next, we made notes of our first impressions, thoughts, and initial analysis. As this process continued, a code was assigned to the key thoughts captured from the transcripts. Codes were then sorted into categories based on how they were related and linked.^14^ To assure that the primary codes were meaningful for the clinician/researcher who was actively involved in developing and testing the MCA tool, in an interactive process, the primary codes and the coding scheme were shared and discussed at each stage until obtaining consensus. NVivo 12 qualitative data management software (NVivo qualitative data analysis software; QSR International Pty Ltd Version 12, 2020) was used later for further data management and to facilitate analysis.

To promote trustworthiness, the research team recorded analytical memos for each session reflecting the group dynamic and researchers’ self-reflection. Getting back to participants to member-check and assure the data accuracy was another measure we used to ensure trustworthiness of the data.^13,14^

The research team included experts with strong backgrounds in bioethics, medicine, nursing, and qualitative methods.

## Ethical considerations

Approval to conduct this study was received from the Providence Health Care/University of British Columbia Behavioural Research Ethics Board (reference number: H16-01858). All participants were provided with an explanatory statement about voluntary participation and provided their written informed consent before the MCA session. We assured participants that the content of the discussion will be confidential, and only the research team will have access to the notes. All study notes were kept in a locked cabinet in the Principal Investigator’s locked office. Only the Principal Investigator (PD) and the qualitative researcher co-investigator (SJ) had access to the notes for the purpose of data analysis.

## Results

The aggregated and categorized responses according to the steps of the MCA were as follows:

### What participants were constrained to do

There were many situations in which ICU staff felt constrained to engage in work that conflicted with their values. These situations related to (a) goals of care, (b) communication, (c) teamwork, (d) respect for patient’s preferences, and (e) managerial system.

#### Goals of care

One of the most morally distressing situations for participants was the situation in which there was ambiguity in goals of care related to “cure or comfort,” and doing too much or too little was perceived to cause more harm than good to the patient. Some participants perceived that a few ICU attending physicians were disinclined to engage in communication processes related to goals of care and that this resistance fueled the ambiguity. In some situations, this ambiguity was perceived to be damaging to both the patient and the health care system overall. For example, one participant talked about a 43 year-old patient who had AIDS, malnourishment, meningitis, and other organ failures. There was no change in his status since he had been admitted to ICU two months earlier. *“None of us know where we are headed with this guy, is this what he wants?”* (Allied Health Care Provider (AHCP))

Some participants mentioned that the care plan was not providing optimal comfort for the patient. Sometimes it is what you can’t do (e.g., not giving medications) that causes moral distress such as *“when patient extubation (was) delayed until the morning, watching a patient seize when medications have been withheld”*. (AHCP)

Participants noted that there was a disconnection between physicians and nursing/allied health regarding goals of care. For example, participants talked about the disconnection between documentation about goals of care, and actual action or practice. They explained how watching someone suffering during reintubation when they knew that the patient was going to die was distressing for them and the patient because they believed, *“it could be* *a lot less suffering”* (RN&AHCP)

#### Communication

Affects both patients and ICU health care providers; ineffective communication has consequences for both. One allied health care provider mentioned *“(There is) a lack of interdisciplinary sharing of the patient’s condition and reasoning for a plan”*. Some nurses also stated that some physicians do not communicate adequately with the family which leads to a lack of understanding for the family members. Reluctance of some ICU attending physicians to engage in communication causes frustration so that participants feel like: *“communication sucks” (Staff)*

Ineffective communication can also erode trust. Participants shared experiences of deteriorating trust among team members and also between team members and family members when they felt constrained from providing information to the family. A nurse said: “*I am keeping quiet. I was not as honest as I should be with the patient because prior communication to family was not clear”. (RN)*

#### Teamwork

Some nurses working in ICUs felt that teamwork was dysfunctional and that their work is not aligned with that of physicians and management*.* Some of them stated: *“there is a disconnection between physicians and nursing/allied health regarding goals of care”. (RN& AHCP)*

In describing a situation, both ICU nurses and other staff believed that *“the physicians have an agenda for themselves, and our assessment is not considered*” *(RN and Staff);* in such situations*, “We don’t feel a part of the team”* (RN and Staff)

Lack of availability and will of the team to address the goals of care emerged from ICU staff statements: *“rotation of attending physicians makes this situation even worse”. (AHCP)*

#### Respect for patients’ preferences

Participants felt morally distressed when there was no informed engagement of the patient/family in the care plan. *“Doing CPR or other invasive measures when the patient-family does not understand the consequences or what it means was an example of those circumstances”*. (RN & Staff)

For example, there was a situation in which the patient was known from another clinical setting to a participant working in the ICU during the night shift. The patient was intubated and based on the report from colleagues, this was not the patient’s wishes. The team was waiting for some clarification before changing the plans. The patient was in pain and could not be sedated or given analgesics for a medical reason.

There were other examples confirming disrespect of patients’ preferences such as when the health care team provided CPR and other aggressive interventions for an unconscious and near-death patient, due to family members’ wish for full resuscitation. According to ICU staff, *“the patient verbally expressed upon his admission to critical care a preference for no pacemaker”.* (AHCP)

Participants perceived restriction in their ability to provide support to patients and families in treatment decisions. They explained witnessing how patients are pushed to accept what the physicians decide; this situation was challenging for ICU staff who have less power.

One of the participants said that she avoided going to the patient’s room because the patient’s wishes were ignored: *“I feel upset about the situations* *in which patients' opinion would not make a difference because the team is just trying to save his life” (AHCP)*

Several participants shared their experience of being involved in disrespect or ignorance of patient’s preferences: *“we were forced to prolong the patient’s life without understanding the wishes of the patient and family, and without the ability to explore the palliative approach”*. (RN and AHCP).

Another nurse: *“not letting someone die when it is inevitable is undignified”* (RN). They were distressed at not being empowered to use available supportive resources to help address and resolve the distressing situations: *“We are not allowed to invite *ethics* and palliative care personnel when we think it might be helpful”*. (RN and AHCP)

Witnessing patient suffering is always distressing for ICU staff who have the experience of watching someone get reintubated when they know that they are going to die. *“It could be a lot less suffering*.*”* (RN) Another participant reported “*witnessing patients suffering when comfort medications are withheld and not giving adequate analgesia during painful procedures” (RN)*

Disrespect to the family and making them feel unsafe was also distressing for the participants. One of the ICU nurses said: *“sometimes I see inadequate privacy and respect for family to express their feelings and concerns during a bedside update, when they don’t want to be overheard by other patients or by the patient themselves”.* (RN)

On the other hand, sometimes hastening death can cause moral distress. A physician explained: “*supervising the withdrawal of life support in anticipation of organ donation in a patient who was not brain dead was distressing*”. *(Physician, in the context of donation after cardiac death)*

#### Managerial system

ICU staff need to feel safe and supported by “the system” while they advocate for patients. However, sometimes staff are aligned with the family but this perspective conflicts with system expectations. One physician mentioned that “*in the process of withdrawing life support as part of organ donation after cardiac death, (his) priority for care was to the family*”, even when this conflicts with expectations of the system. (Physician)

Some organizational issues lead to staff feeling devalued. For example, one of the nurses explained: *“they (upper management) are sending us to take assignments in other units, and being used as the float pool for the hospital. The staff do not choose to work in those areas; they also feel unsafe and distressed about this policy, but their (management) response is that a nurse is a nurse.”* (RN) In one unit, the participant explained how eight nurses had left this hospital due to re-deployment in just a few months. Now having a total of 13 vacant nursing shift sequences in one ICU, the remaining staff felt devastated. The inexperience of many remaining staff led to concerns about unsafe coverage at times.

### The effects of moral distress on intensive care unit staff

Moral distress affected ICU staff in different ways. Although physical problems such as headaches, gastric discomfort, loss of sleep, and muscle spasms were mentioned by some participants, emotional and mental problems were reported most commonly. Some participants explained how they felt out of place, how their mind was pre-occupied so that they were unable to think straight and felt disconnected with their role. They also had strong feelings of guilt, not being able to accomplish what they set out to do and questioning their own practice. Some of the most common expressions of those effects were feeling angry, annoyed, anxious, ashamed, avoidant, discouraged, exhausted, fatigued, frustrated, guilty, irritable, helpless, hopeless, and sad.

### Participants’ responses to moral distress

#### Self-care measures

Included both active and avoidant strategies. Active strategies were those that protected them from the effects of moral distress and could help them to be stronger and to cope better. These included doing outdoor activities and exercise, dance, yoga, socializing with loved ones, enjoying good food and drink, and having time alone for self-reflection. They also used avoidant coping strategies such as removing themselves from the environment and walking out of the unit, avoiding the situation, changing work status to part-time, not taking night shifts and calling in sick, to try to temporarily mitigate the moral distress.

#### Communication

Strategies included communicating with colleagues and the patient’s family concerning the specific issue, sharing the problem with their colleagues, clarifying the plan, trying to find common ground, and bringing the issue up for discussion at multidisciplinary rounds.

#### Seeking support

Strategies included taking the problem to a higher level by looking into existing ICU guidelines, getting support from the nurse manager, meeting with senior managers and attending physicians, talking to administrators, and consulting with risk managers and ethicists.

### Participants’ core values, self-interests, and self-realizations that are either in conflict or are consistent with the situation that is associated with moral distress

Core values played a significant role in the conflicts that caused moral distress. Participants believed that the behaviour they were constrained to do conflicted with their values of respecting and supporting patient autonomy, promoting patient well-being (beneficence), minimizing harm (non-maleficence), avoiding unnecessary pain and suffering (such as by providing futile treatment), trustworthiness and honesty, human dignity, justice, and safety.

Self-interests were also “actors” in these conflicts. For example, doing their job, performing their duty, and being a team player in ICU were consistent with the behaviors that they were constrained to do. Self-realization (identity) issues that conflicted with the behaviors that they were constrained to do included independence, tolerance, collaboration, patient advocacy, delivering high-quality care, respectful communication, and keeping their job meaningful.

### Existing factors that help or hinder the situations that are associated with moral distress

Although participants reported both helping and hindering factors, hindering factors were given a higher semi-quantitative weight. Hindering factors were: inconsistency in the care plan, lack of resources, administrative barriers, ineffective communication, lack of support, lack of information and education, and ICU culture. Helping factors were mostly related to communication, teamwork, and the available support resources. An inclusive approach and consistent care team including the family physician, and bedside nurse involvement in the patient and family meetings and rounds were examples that enhanced transparency and facilitated dialog. Some participants explained situations in which informal conversations with the family about the patient’s status and the goals of care, followed by listening and responding to their concerns helped to build trust and clarify expectations, which alleviated the moral distress in the unit.

### Participants’ new responses to situations that are associated with moral distress (after participating in moral conflict assessment)

After working through the MCA and reviewing all of their responses to this point, participants were asked to suggest potential new responses to situations that are associated with moral distress. The most common response in this subcategory was improving communication, both among health care providers and between health care providers and family members. Education and training about death and dying, difficult conversations, advance directives, palliative approach (prevention and relief of suffering of individuals who have life-limiting conditions by early identification, assessment, and treatment of pain, and by addressing physical, psychosocial, and spiritual needs of the patient and their family members), patients' rights, and teamwork were also suggested for health care providers working in ICUs. In addition, education regarding advance care planning, goals of care, palliative approach, patients' rights, ICU services and admission criteria, end-of-life options, updates about patients' conditions, and preparing for adverse outcomes was suggested for patients, family members, and other citizens.

### Observable changes after participating in the moral conflict assessment

Participants expressed satisfaction of having a neutral third party to help them articulate what was at the heart of the issue. They explained that going through the MCA brought the problem to light and helped them to identify and analyze the components of the moral conflict, enabling them to see possible concrete ways to improve the situation. Participants stated that staff satisfaction, education and training, teamwork, communication with colleagues, patients, and families, and use of the palliative approach were some of the measures of success that could be achieved by reflecting on moral distress.

There were also some immediate effects of the MCA that made them feel better about the situation. One nurse said:

*“Having people understand what I have been going through—helps.” (RN)* Other nurses echoing the same *feelings* said*: “It was a good opportunity to have their perspective heard*”. (RN)

And: *“I liked thinking through the issue and reframing how advance care planning and other interventions might help address similar situations in the future”*. (RN)

Having the issues summarized and reflected back to them seemed to help*.* *“It helped me problem-solve to make me feel better about the situation, and hopefully improve the situation” (AHCP)*

*“This was a reflection about moral distress and a good way to think about possible solutions. It is nice to step back from a problem and ruminate out-loud and recognize why I get angry. It also helps to know what I need to focus on to help to clarify thoughts and be immediate in future responses”* (RN)

Some explained that it was calming for them. *“*I feel less distressed about actions that are taken, and feel better knowing that I am part of making an improvement and what feedback I have is important*”* (RN). Others said: *“*This reflection made us feel heard and validated. We feel relieved and less stressed*”* (RN&AHCP) Another participant stated that *“this helps put the pieces of the conflict together”, so “we can see that there are concrete ways to improve the situation”* (Pharmacist)

## Discussion

Using the MCA tool helped the participants to reflect on their feelings in relation to moral distress and to arrive at new potential solutions. Our participants characterized the causes of moral distress by describing the situations in which they are constrained from doing what they consider to be the correct moral action. These findings are corroborated by previous studies.^4,9,16–18^ However, providing logical and context-based solutions, to prevent and alleviate moral distress, are some of the novel findings in this study.

The effects of moral distress as described by our participants were both physical and psycho-emotional, but more often the latter. In their qualitative study, Shoorideh et al. found pain, digestive and sleeping disorders, fatigue, and energy reduction to be some physical reactions to moral distress.^17^ Henrich et al. reported that ICU staff show emotional responses such as frustration, feeling embarrassed, and discouraged when their concerns were ignored.^7^ This is aligned with the psycho-emotional effects that our participants articulated such as feeling anxious, ashamed, avoidant, discouraged, fatigued, exhausted, frustrated, hopeless, sad, and not being able to accomplish what they set out to do. These reactions have been reported in other nursing studies^10,18,19^ and have consequences for both ICU staff and the health care system.

Our participants reported using both productive and avoidant coping strategies in response to moral distress. Productive strategies such as communication with colleagues and getting support have been mentioned in other studies and professional recommendations.^2,19,20^ Using productive self-care strategies is recommended for nurses and other health care providers to be able to move towards moral resilience. Specifically, Rushton et al. emphasize nurses’ obligation to address their own suffering.^19^ Avoidant strategies such as being less invested in their work and attempting to distance themselves from the distressing situations, have also been described previously.^7^ However, quitting their job, as a response to moral distress, particularly for nurses^7,8,20,21^ was not stated by our participants. Despite this, quitting work must be acknowledged as a latent negative outcome for the health care system.

Our participants explained factors that can help or hinder moral distress and how they would respond to moral distress in the future. The new responses suggested by the participants were simple and feasible interventions that did not require a lot of resources or time, but according to the participants, could make a huge difference in the level of moral distress that they experienced. For example, improving communication and providing education for both health care providers and members of the public are small changes that can have positive effects such as improved staff satisfaction and teamwork. Using or even considering the palliative approach, when appropriate, is another strategy that could address a common source of moral distress.^22^ After participants participated in the MCA, and articulated new responses to situations that cause moral distress, they generally had positive attitudes. Specifically, they may have felt empowered to exercise their moral agency,^23^ in contrast to feeling disempowered and not being able to do anything, which is associated with broader moral costs^24^ for health care providers. Rushton et al. have also recommended specific collaborative or team-based interventions, which may improve nurse–physician communication through discussions that explore their differing perspectives and role expectations.^19^

Recommendations from this research differ from other studies in that they arise from clinicians as a potential new response to an actual situation that they have experienced. One of the most important features of the MCA is that participants not only address the causes and consequences of moral distress in general, but also link them with specific potential feasible solutions. This approach highlights innate skills within a clinician group, empowering team members themselves to deal with stressful situations resulting from moral distress. An additional advantage of the MCA method, using an “external” facilitator if necessary, is the ability to address critical incidents in real time rather than in a “postmortem” manner.

Study participants reported feeling heard and understood and subsequently relieved after the sessions. They also explained how reflecting on their experiences of morally distressing situations actually empowered them to step back from the problem and think about possible solutions. This phenomenon can also be linked to moral agency which implies self-directed capacity or choice to act.^25^ Establishing an alternative “story” about a morally distressing situation can help clinicians to shift their perspective from being a victim to being an empowered agent^26^ and can help to transform their moral distress into resilience.^19^

Strengths of this study include the multidisciplinary representation and engagement by ICU personnel and interprofessional research team. Furthermore, we used a new participatory approach for MCA that assesses the causes and consequences of moral distress for ICU staff and provides the opportunity to generate new strategies to resolve the problem. Limitations of this study include dominant representation of nurses, and participants from 3 ICUs in one healthcare region. These findings may not be generalizable to other staff in other settings. In addition, there was no audio or video recording of the MCA sessions, which may have allowed for more quotations to analyze. However, considering the types of responses solicited from participants (single words or short phrases), and the fact that our reports, including quotations, were verified by the original participants, it is unlikely that we have missed important content.

## Conclusion

We found that characterizing moral distress in this systematic way helped the ICU staff to gain a better understanding of the problem and find new strategies to resolve the problem by using more productive coping strategies. ICU staff acknowledged that participating in the MCA helped them to reflect on their own thoughts and use their strengths to create capacity for turning a distressing situation into a learning opportunity and to improve the situation as a moral agent. Therefore, this tool can be adopted widely by health care personnel in a variety of settings throughout the world as a strategy to address moral conflict.
